# Engineering HIV-Resistant Human CD4+ T Cells with CXCR4-Specific Zinc-Finger Nucleases

**DOI:** 10.1371/journal.ppat.1002020

**Published:** 2011-04-14

**Authors:** Craig B. Wilen, Jianbin Wang, John C. Tilton, Jeffrey C. Miller, Kenneth A. Kim, Edward J. Rebar, Scott A. Sherrill-Mix, Sean C. Patro, Anthony J. Secreto, Andrea P. O. Jordan, Gary Lee, Joshua Kahn, Pyone P. Aye, Bruce A. Bunnell, Andrew A. Lackner, James A. Hoxie, Gwenn A. Danet-Desnoyers, Frederic D. Bushman, James L. Riley, Philip D. Gregory, Carl H. June, Michael C. Holmes, Robert W. Doms

**Affiliations:** 1 Department of Microbiology, University of Pennsylvania School of Medicine, Philadelphia, Pennsylvania, United States of America; 2 Sangamo BioSciences, Richmond, California, United States of America; 3 Department of Medicine, University of Pennsylvania School of Medicine, Philadelphia, Pennsylvania, United States of America; 4 Divisions of Regenerative Medicine and Comparative Pathology, Tulane National Primate Research Center, Tulane University School of Medicine, Covington, Louisiana, United States of America; 5 Abramson Family Cancer Research Institute, University of Pennsylvania School of Medicine, Philadelphia, Pennsylvania, United States of America; University of Zurich, Switzerland

## Abstract

HIV-1 entry requires the cell surface expression of CD4 and either the CCR5 or CXCR4 coreceptors on host cells. Individuals homozygous for the *ccr5Δ32* polymorphism do not express CCR5 and are protected from infection by CCR5-tropic (R5) virus strains. As an approach to inactivating CCR5, we introduced CCR5-specific zinc-finger nucleases into human CD4+ T cells prior to adoptive transfer, but the need to protect cells from virus strains that use CXCR4 (X4) in place of or in addition to CCR5 (R5X4) remains. Here we describe engineering a pair of zinc finger nucleases that, when introduced into human T cells, efficiently disrupt *cxcr4* by cleavage and error-prone non-homologous DNA end-joining. The resulting cells proliferated normally and were resistant to infection by X4-tropic HIV-1 strains. CXCR4 could also be inactivated in *ccr5Δ32* CD4+ T cells, and we show that such cells were resistant to all strains of HIV-1 tested. Loss of CXCR4 also provided protection from X4 HIV-1 in a humanized mouse model, though this protection was lost over time due to the emergence of R5-tropic viral mutants. These data suggest that CXCR4-specific ZFNs may prove useful in establishing resistance to CXCR4-tropic HIV for autologous transplant in HIV-infected individuals.

## Author Summary

For HIV to enter T cells, the virus first binds to a primary surface receptor CD4 and then to a coreceptor, either CCR5 or CXCR4. Previously we engineered zinc-finger nucleases (ZFNs) to specifically disrupt the CCR5 gene in primary human T cells, the predominant cell type infected and killed by HIV. This makes the cell permanently resistant to CCR5-tropic HIV; however, viruses that can utilize CXCR4 can still infect cells. ZFNs function as molecular scissors that cut a specific region of DNA. Then, the cell's own machinery repairs this cut, often introducing mutations that result in a non-functional protein. Currently, a clinical trial is underway in which HIV-infected individuals' own cells are removed from their blood, treated with the CCR5-ZFNs, and then infused back. Here, we report the use of novel zinc-finger nucleases that specifically and permanently disrupt the CXCR4 gene in T cells. This treatment results in resistance to CXCR4-tropic HIV. In addition, we combine CXCR4 and CCR5 genetic disruption to make cells resistant to all strains of HIV. Our long-term goal is to engineer HIV-resistant CD4+ T cells in infected individuals that can be reinfused and hopefully enable them to control infection in the absence of anti-viral drugs.

## Introduction

For HIV to infect cells, the viral envelope (Env) protein must bind to the host protein CD4 and then to a coreceptor, most commonly CCR5 (R5 HIV) (reviewed in [1]). The importance of CCR5 for HIV-1 pathogenesis is shown by the fact that individuals who are homozygous for an inactivating 32 base pair deletion in *ccr5* (*ccr5Δ32*) are highly resistant to HIV infection [2], [3], while heterozygotes typically live longer after HIV infection due to reduced CCR5 expression levels [4], [5]. Recently, an HIV infected patient with acute myelogenous leukemia received a bone marrow transplant from a *ccr5Δ32* homozygous donor [6]. This patient's viral load remains undetectable even in the absence of anti-retroviral therapy more than three years post-transplant, suggesting that this individual's HIV infection has been eradicated. In theory, the success of this approach could be recapitulated by inhibiting CCR5 with an orally bioavailable small molecule such as maraviroc, which binds to CCR5 and prevents its use by most R5 HIV-1 strains. However, virus strains that can utilize CXCR4 either in place of (X4 HIV) or in addition to CCR5 (R5X4 HIV) are found at significant levels in roughly 50% of late-stage infected individuals [7], [8], supporting the need for therapies targeted to CXCR4 [9]. Ideally, an approach to target CXCR4 would complement CCR5-specific therapy, but the broad expression pattern of CXCR4 has made systemic inhibition of this coreceptor by small molecules problematic [10], [11]. In addition, resistance to CCR5 and CXCR4 antagonists can arise in patients by mutations in the viral envelope protein that enable it to utilize the drug-bound forms of these coreceptors [12]–[16]. The ability of HIV-1 to adapt to new selective pressures and the plasticity with which Env interacts with its coreceptors argues for approaches that reduce or eliminate coreceptor expression rather than simply altering coreceptor conformation. If approaches could be developed that specifically target expression of both CCR5 and CXCR4 on CD4+ T cells, virus entry should be inhibited more effectively.

**Figure 1:**
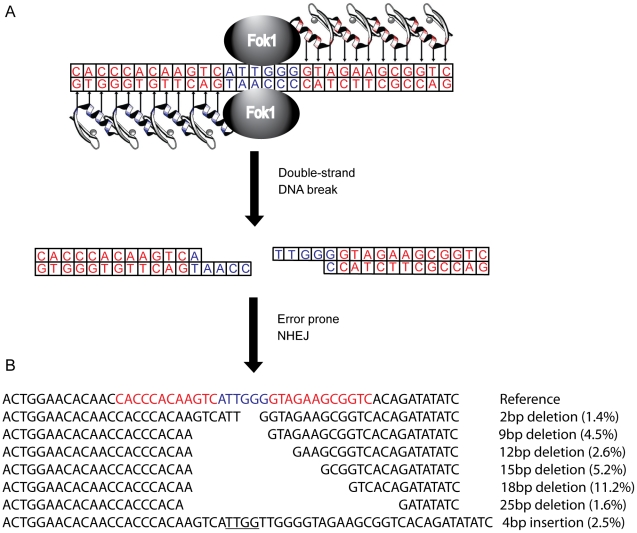
Zinc finger nucleases (ZFNs) bind, cleave, and disrupt *cxcr4*. (A) A CXCR4-specific ZFN pair was generated, comprised of two DNA-binding zinc finger proteins (ZFPs) each fused with a *FokI* endonuclease monomer. Each ZFP was designed to target 12 bp of *cxcr4* sequence (in red), separated by 6 bp (in blue), conferring 24 bp of total specificity. Upon binding of both ZFPs, the *FokI* domains can dimerize and cleave the double stranded DNA. The subsequent double strand break is then repaired by error prone non-homologous end-joining resulting in various targeted mutations and a non-functional protein product. (B) The most common mutations induced by the X4-ZFNs, as detected by 454 deep sequencing, are indicated with their frequencies among all ZFN-induced lesions. In-frame deletions were preferentially generated with the most common being an 18 bp deletion, referred to as CXCR4Δ18. Frequences were averaged across five independent experiments in the absence of HIV infection.

Several genetic approaches have been taken to reduce or eliminate CCR5 expression in human cells, including the use of ribozymes [17], [18], single-chain intracellular antibodies [19], trans-dominant coreceptor mutants [20], and RNAi [21], [22]. However, these studies are limited by the requirement for stable expression of an exogenous gene. To circumvent this, a CCR5 specific zinc-finger nuclease pair (R5-ZFNs) has been developed [23]. Zinc finger proteins that recognize a specific 24 bp DNA sequence are fused with a monomeric cleavage domain from *FokI* endonuclease that functions only as a dimer (Figure 1). For DNA cleavage to occur, two zinc finger proteins must bind, each to specific, adjoining sequences in the CCR5 gene, leading to *FokI* dimerization and subsequent DNA cleavage resulting in a double strand break [24]–[26]. The double strand break then can be repaired by error-prone non-homologous end joining (NHEJ) often introducing insertions and deletions leading to a non-functional gene product when this break is placed within the coding region of the targeted gene [27]. Following introduction into human CD4+ T cells [23] or hematopoietic stem cells [28] via an adenovirus vector or DNA nucleofection, respectively, the *ccr5* gene was efficiently and specifically disrupted. This confers protection *in vitro* and in humanized mice to infection by HIV-1 isolates that require CCR5 (but not CXCR4). Several early stage clinical trials using autologous infusions of ZFN-generated CCR5-modified CD4+ T cells are currently underway (clinicaltrials.gov identifiers NCT00842634, NCT01252641, NCT01044654).

In this study we describe the design and pre-clinical evaluation of a CXCR4-specific ZFN pair (X4-ZFNs) that specifically and efficiently disrupts *cxcr4*, rendering human CD4+ T cells permanently resistant to HIV-1 strains that require CXCR4 for infection. We also demonstrate that *cxcr4* can be safely and efficiently disrupted in CD4+ T cells obtained from *ccr5Δ32* homozygotes resulting in cells resistant to all strains of HIV-1 tested. This suggests that combined treatment of mature CD4+ T cells with X4-ZFNs and R5-ZFNs can provide permanent protection against HIV-1 infection.

## Methods

### Zinc-finger nuclease constructs

We designed ZFNs specific to the human and rhesus CXCR4 and CCR5 genes using a previously described approach [29]. One ZFN pair was used to target both the human and rhesus macaque CXCR4 genes since the 24 bp target sequences are identical. Zinc-finger proteins were optimized against the target gene sequence and assembled as described [30] from an archive of *in-vitro-*selected modules [31], [32]. The ZFP moieties (target gene; ZFP name; target sequence (5′→3′); recognition α-helices (finger number)) are as follows: CXCR4; X4-ZFN-L; GTAGAAGCGGTC, DRSALSR (1), RSDDLTR (2), QSGNLAR (3), QSGSLTR (4); CXCR4; X4-ZFN-R; GACTTGTGGGTG, RSDSLLR (1), RSDHLTT (2), RSDSLSA (3), DRSNLTR (4). Rhesus CCR5; rhR5-ZFN-L; GATGAGGACGAC, RSDNLAR (1), TSGNLTR (2), RSDNLAR (3), TSGNLTR (4); Rhesus CCR5; rhR5-ZFN-R; AAACTGCAAAAG; RSDNLSV (1), QKINLQV (2), RSDVLSE (3), QRNHRTT (4)., The human CCR5-specific ZFNs are described in Perez et al [23]. The Ad5/F35 adenoviral vectors were generated on an E1/E3 deleted backbone. The ZFNs targeting either the *cxcr4* or *ccr5* genes were linked via a 2A peptide sequence and cloned into the pAdEasy-1/F35 vector under control of the CMV TetO promoter, and the Ad5/F35 virus for each construct was generated using TREx 293T cells as described [33]. The Ad5/F35 vector encoding the X4-ZFNs is identical to that use by Nilsson, et al. [33] except for the ZFN inserts, promoter, polyA and linker sequences.

### Cel1 (surveyor nuclease) assay

Genomic DNA was extracted with the MasterPure kit (Epicentre Biotechnologies) according to manufacturer's instructions. Frequency of gene modification by NHEJ was evaluated as described previously [23], [25], [28]. Briefly, the purified genomic DNA was used as a template to amplify a fragment of the *cxcr4* gene using the specific primers (human CXCR4: 5′-CAACCTCTACAGCAGTGTCCTCATC -3′and 5′- GGAGTGTGACAGCTTGGAGATG -3′; rhesus CXCR4: 5′- GGTGGTCTATGTTGGAGTCTGG -3′and 5′- GGAGTGTGACAGCTTGGAGATG -3′) in the presence of a ^32^P-dATP and dCTP. The PCR products were then heated, allowed to re-anneal followed by treatment with the mismatch-sensitive Surveyor nuclease as described in order to detect insertions and deletions caused by NHEJ. For humanized mice samples, whole genome amplification using the REPLI-g Mini Kit (Qiagen) was conducted prior to the surveyor nuclease assay due to limiting cell numbers.

### Human CD4+ T cell stimulation and transduction

Fresh CD4+ T cells from normal human donors, purified by negative selection, were obtained from the Center for AIDS Research Human Immunology Core at the University of Pennsylvania. 2.5 million CD4+ T cells were seeded at a density of 0.8×10^6^ cells/ml in RPMI containing 10% fetal calf serum, 1% penicillin/streptomycin, and 100 U/ml interleukin-2 (IL-2). The cells were stimulated with anti-CD3/anti-CD28 coated magnetic beads at a 3∶1 bead to cell ratio [34]. Approximately 18 hrs post-stimulation, the cells were transduced with an Ad5/F35 vector encoding either the X4-ZFNs or R5-ZFNs at a multiplicity of infection (MOI) of 600. Beginning 72 hours post-stimulation, cells were counted every 48 hours using trypan blue dye exclusion on an automated hemocytometer (Countess, Invitrogen) and split to 0.8×10^6^ with fresh media containing 100 U/ml IL-2. Five days post-stimulation, the magnetic beads were removed and washed twice in fresh media. Cells were counted and split until cell growth plateaued 10–14 days post stimulation. For longer experiments, cells were restimulated with beads and cultured for an additional 10–14 days.

### *In vitro* HIV-1 challenge of CD4+ T cells treated with AdX4-ZFNs

Five days post-stimulation the anti-CD3/anti-CD28 coated magnetic beads were removed from each of the three cultures (non-transduced (NTD), AdX4-ZFNs, and AdR5-ZFNs) and 2.5 million cells were seeded in each of four cultures that were subsequently infected with either Bk132 (primary X4 isolate), HxB2 (lab-adapted X4 isolate), R3A (R5X4 primary isolate), or media only (mock). 100 ng p24 of HIV-1 was used per million cells.

### Flow cytometry

All staining was done at room temperature in FACS Wash Buffer (1 mM EDTA, 2.5% fetal calf serum in PBS) and all antibodies were from BD Biosciences unless otherwise noted. 0.5–1.0×10^6^ cells were washed in PBS and stained with Live/Dead Aqua (Invitrogen) for 10 min. Then, anti-CD4 PE Cy5.5 and anti-CXCR4 APC (clone 12G5) were added and cells were stained for 20–30 minutes. Cells were then washed and permeabilized per manufacturer's protocol using Cytofix/cytoperm (BD) and stained intracellularly for HIV gag with KC57-RD1 (Beckman Coulter). For compensation, ArC beads (Invitrogen) were used for live/dead, and CompBeads (BD) were used for all other fluorochromes. To detect wtCXCR4 and CXCR4Δ18 in 293T transient transfection experiments, anti-CXCR4 APC (clone 12G5) and anti-CXCR4 PE (clone 4G10) (Santa Cruz Biotechnologies) were used. All samples were run on an LSRII (BD) and analyzed using FlowJo 8.8.6 (Treestar Inc).

Events were gated as follows: singlets (FSC-A by FSC-H), live cells (SSC-A by Live/Dead), lymphocytes (FSC-A by SSC-A), CD3+CD4+ (CD3 by CD4), and then events were divided into CXCR4+ and CXCR4- populations based upon a fluorescence minus one (FMO) control.

### 454 deep sequencing and *cxcr4* analysis

Genomic DNA was isolated from CD4+ T cells using the QIAamp DNA Micro Kit (Qiagen). For each condition, 200 ng genomic DNA was then PCR amplified using Platinum Taq High Fidelity (Invitrogen) using the following primers plus 454 adaptor sequences and 8 letter DNA barcodes: CAACCTCTACAGCAGTGTCCTCATC (forward) and GGAGTGTGACAGCTTGGAGATG (reverse). Cycle conditions were 95° for 5 min, then 30 cycles of 95° for 30 sec, 55° for 3 sec, 68° for 30 sec, followed by 68° for 2 min. Following PCR amplification the PCR product was analyzed on a 2% agarose gel and then extracted and gel purified using Wizard SV Gel and PCR Clean-Up System (Promega). Quant-iT dsDNA High-Sensitivity Assay Kit (Invitrogen) was then used to determine the concentration of each bar-coded amplicon. DNA samples were then pooled at an equimolar ratio and run on a Roche/454 GS FLX using standard chemistries at the University of Pennsylvania's DNA Sequencing Facility. Approximately 30,000–100,000 reads were obtained for each experiment. CXCR4 pyrosequencing data were assigned to samples by DNA barcode. Any reads containing ambiguous base calls or without a perfect match to barcode and primer were discarded. All remaining reads were aligned to the CXCR4 reference sequence using Mosaik (http://bioinformatics.bc.edu/marthlab/Mosaik). All deviations from the CXCR4 consensus sequence 40 base pairs up or downstream from the ZFN binding site were determined. Any reads that did not extend across this region or that failed to align were discarded. Reads containing only two or fewer substitutions were not classified as mutations as these likely represent sequencing artifacts. Next, background pyrosequencing error, identified by an untransduced control sample, was subtracted from each group of reads. For frameshift analysis, the sequencing error was determined and subtracted for each individual insertion or deletion size.

To ensure sufficient sampling of diverse amplicons, at least 200 ng gDNA was used for CXCR4 analysis and at least 400 ng gDNA was used for off-target site amplification, representing the genomic DNA content of approximately 70,000 and 140,000 alleles, respectively. Determining genetic disruption frequency by both the Cel1 and 454 assays require the assumption that wild type and disrupted alleles are not differentially amplified.

### Systemic evolution of ligands by exponential enrichment (SELEX) and determination of off-target sites

To empirically determine the DNA binding preference of the X4-ZFNs, we employed SELEX as previously described [23]. Briefly, each ZFP was HA-tagged and incubated with randomized DNA oligonucleotides and anti-HA Fab fragments. Any DNA bound to the ZFPs was then isolated and amplified. The newly amplified DNA was then used to repeat this process for a total of four rounds of enrichment. The DNA pool was then sequenced at approximately 50× coverage to generate a positional-weighted matrix. This matrix was then aligned to the human genome with the following criteria: putative off-target sites could have up to six mismatches compared to the SELEX consensus sequence, the ZFP pairs must be separated by either 5 or 6 bps, and both ZFP homo- and heterodimers were considered. Off-target sites were ranked and scored by multiplying the probability of each nucleotide at each of the 12 positions of the positional-weighted matrix. The highest scores were then deemed most likely to be disrupted. 454 off-target site data was analyzed as discussed previously [23].

### NSG mice

NSG (NOD.Cg-*Prkdc^scid^Il2rg^tm1Wjl^/*Szj) mice, 8–9 weeks old at time of initial injection, were derived from breeders purchased from The Jackson Laboratory (Bar Harbor, ME). Animals were maintained in a defined flora animal barrier facility at the University of Pennsylvania's Stem Cell and Xenograft Core.

Human CD4+ T cells were isolated and stimulated as previously described and then transduced with an Ad5/F35 vector expressing either the R5-ZFNs or the X4-ZFNs at an MOI of 600. Cells were maintained as previously described. Ten days post stimulation 10^7^ modified cells resuspended in 100 µL PBS were injected intravenously into the tail vein of each mouse. 23 animals received cells treated with X4-ZFNs and 22 mice received cells treated with R5-ZFNs. Animals were randomized by age, sex, and cage. Mice were maintained on the antibiotic Baytril (Bayer) for 24 hours post-injection.

To infect the mice with HIV-1, 10^5^ autologous CD4+ T cells previously infected with X4 HIV-1 strain Bk132 were injected into the tail vein of each mouse. Autologous cells used to infect mice that were not transduced were obtained and stimulated simultaneously as the initially engrafted cells. Five days post-stimulation cells were infected with 100 ng p24/million cells and then were cryopreserved four days post-infection. Cell engraftment was assessed 27 days post injection, and mice were infected with HIV-1 the following day.

To obtain whole blood, mice were anesthetized with isoflurane and a capillary tube was used to drain the retroorbital vein. Human CD4+ T cell counts were determined by staining 50 µl of whole blood in Trucount tubes (BD) with anti-CD45 FITC (Biolegend), anti-CD3 Qdot 655 (Invitrogen), anti-CD4 Alexa Fluor 700, anti-CD8 Pacific Blue (Biolegend), and anti-CXCR4 PE-Cy5. Human CD4+ T cells were defined as CD45+CD3+CD4+CD8-.

At the time of sacrifice, a cardiac puncture was performed to obtain maximal blood volume and then the spleen was harvested. Spleens were homogenized and erythrocytes were lysed with ACK lysis buffer (Invitrogen) before cell purification. Human CD4+ T cells were then isolated with the Human CD4 Positive Selection Kit using the Robosep robotic cell separator (Stem Cell Technologies).

### Rhesus macaque CD4+ T cell modification

Whole blood from rhesus macaques (Macaca mulatta) housed at the Tulane National Primate Research Center was used for CD4+ T cell isolation and ZFN treatment. Peripheral blood mononuclear cells were isolated by centrifugation with 96% Ficoll (BD), followed by erythrocyte lysis with ACK lysis buffer. CD4+ T cells were then isolated by negative selection with a non-human primate CD4+ T cell selection kit (Miltenyi). Cells were then stimulated with 1∶4 anti-CD3 (clone FN-18)/anti-CD28 (clone L293) M-450 tosylactivated beads (Invitrogen) at a ratio of 1 bead per cell [35], [36].

Approximately 18 hours post-transduction, cells were transduced with an Ad5/F35 vector expressing either the X4-ZFNs or rhesus specific R5-ZFNs. Cells were maintained in culture as human CD4+ T cells. Surveyor nuclease assay was performed six-ten days post transduction to assess disruption efficiency.

### Ethics statement

Human CD4+ T cells were obtained after written informed consent and approval by the University of Pennsylvania's institutional review board. All humanized mouse experiments were approved by the University of Pennsylvania's Institutional Animal Care and Use Committee (Protocol 802436), and were carried out in accordance with recommendations in the Guide for the Care and Use of Laboratory Animals of the National Institutes of Health. All rhesus macaque experiments were approved by the Tulane Institutional Animal Care and Use Committee approval (Protocol P0085; Project 3520) The Tulane National Primate Research Center (TNPRC) is an Association for Assessment and Accreditation of Laboratory Animal Care accredited facility (AAALAC #000594). The NIH Office of Laboratory Animal Welfare assurance number for the TNPRC is A3071-01. All clinical procedures, including administration of anesthesia and analgesics, are carried out under the direction of a veterinarian. Blood was collected while the animals were anesthetized with Tiletamine-zolazepam with Burprenorphine given as an analgesic. All possible measures are taken to minimize discomfort of all the animals used in this study. The University of Pennsylvania and Tulane comply with NIH policy on animal welfare, the Animal Welfare Act, and all other applicable federal, state and local laws.

## Results

### Design and characterization of X4-ZFNs

To genetically disrupt the CXCR4 allele, we designed a pair of zinc-finger proteins (ZFPs) targeting the region of the *cxcr4* gene that encodes residues Asp 187 to Val 196 in the second extracellular loop (ECL2) of this seven-transmembrane domain receptor using methods previously described [29]–[32] (Figure 1). The ECL2 was chosen because this region is less well conserved amongst the CXC family of chemokine receptors, which should reduce the frequency with which other CXC receptors might be targeted, and because ECL2 is important in supporting interactions with the HIV-1 Env protein [37], [38]. Two ZFPs were designed to bind each of two 12 bp targets separated by 6 bp in this region of CXCR4. Each ZFP was then fused to a modified *FokI* cleavage domain, active preferentially as a dimer to reduce nonspecific DNA cleavage, resulting in zinc-finger nucleases (ZFNs) [25]. Upon binding of both X4-ZFNs, the *FokI* nuclease cleavage domains dimerize and then generate a double strand break that can subsequently be repaired by error-prone NHEJ resulting in mutations targeted to the cleavage site that can include missense mutations, deletions and insertions (Figure 1).

### Efficiency of CXCR4 allele disruption in human CD4+ T cells

**Figure 2:**
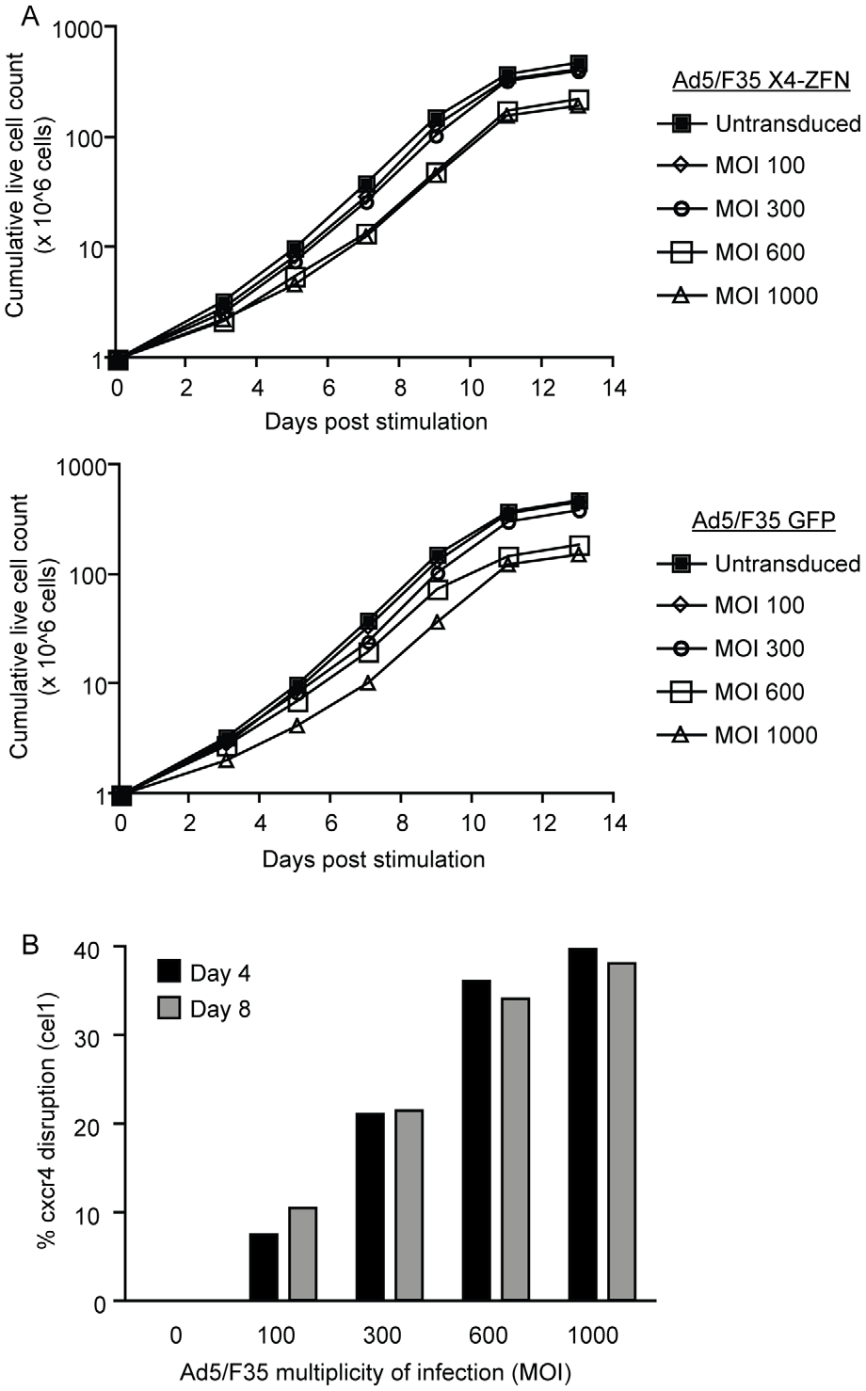
X4-ZFNs mediated disruption of *cxcr4* in primary human CD4+ T cells. (A) Primary human CD4+ T cells were stimulated and transduced with an Ad5/F35 vector expressing either the X4-ZFNs (top) or GFP (bottom) at MOIs from 100–1000. Total live cells were counted at different times after stimulation, and compared to an untransduced control. Data is from one of two independent experiments. (B) *Cxcr4* disruption was determined four and eight days post treatment with the X4-ZFNs by the surveyor nuclease assay (cel1).

To determine the efficiency and specificity with which the *cxcr4* genes could be disrupted in human T cells, we produced a bicistronic Ad5/F35 vector to deliver the X4-ZFNs (AdX4-ZFNs). The Ad5/F35 vector is a serotype 5 virus with the fiber protein from a serotype 35 adenovirus that utilizes CD46 for entry as opposed to the coxsackie and adenovirus receptor (CAR), which is poorly expressed on human CD4+ T cells [39]. Primary human CD4+ T cells were stimulated with anti-CD3/anti-CD28 coated magnetic beads and transduced 18 hours later with AdX4-ZFNs, AdR5-ZFNs which expresses previously described CCR5-specific ZFNs [23], or an Ad5/F35 vector that expresses green fluorescent protein (AdGFP). To identify optimal disruption conditions, multiplicities of infection ranging from 100 to 1000 were employed. Cell growth was monitored every 48 hours post-stimulation for approximately two weeks and the efficiency of CXCR4 disruption was assessed at day five post-transduction by both the Surveyor nuclease assay and by deep-sequencing of the CXCR4 target site. As shown in Figure 2A, the Ad5/F35 vectors had a slight dose-dependent impact on cell growth at higher multiplicities of infection that was similar with the AdX4-ZFNs and AdGFP vectors.

*Cxcr4* allelic disruption efficiencies as determined by either deep sequencing or the Surveyor nuclease assay were comparable, and were approximately 10% at an MOI of 100, 20% at an MOI of 300, 34% at an MOI of 600, and 38% at an MOI of 1000 (Figure 2B). For subsequent experiments we used an MOI of 600 as this provided near-maximal disruption efficiency with limited impact on cell growth. Notably, this is also the MOI being used in an adoptive therapy phase I clinical trial with R5-ZFNs. Importantly, the level of *cxcr4* disruption in cells from multiple donors was stable over nearly four weeks in culture (Table S1), indicating that CXCR4-disrupted cells continued to grow normally. Cell proliferation remained dependent on stimulation, and transformation has not been observed after treatment with ZFNs (data not shown).

### Mutations introduced by cleavage with X4-ZFNs

Deep sequencing of the ZFNs target site 10 days after transduction made it possible to assess the mutations introduced by NHEJ reactions following cleavage with X4-ZFNs. Of the nearly 50,000 modified *cxcr4* alleles analyzed across five independent experiments, 81.1% (range 75.3–81.7%) contained pure deletions from 1–64 bp in size with the most common deletions being 2, 9, 12, 15, 18, and 25 bp, while 13.5% (range 12.8–16.9%) of *cxcr4* alleles contained pure insertions ranging from 1 to 69 bp with more than 90% being 7 bp or less (Figure 1B). The remaining 5.3% (range 4.3–7.4%) of disruption events contained multiple insertions and deletions that may be due to more extensive DNA end-processing or multiple cycles of ZFN-mediated cleavage and subsequent NHEJ. Surprisingly, frameshift mutations occurred at a ratio of 0.90 in-frame per out-of-frame mutation as opposed to the expected frequency of 0.50 (1 in-frame per 2 out-of-frame mutations; Table S1). This unexpected bias likely resulted from microhomology-mediated joining that produced in-frame deletions. To our knowledge, preferential in-frame repair has not been reported or seen with other ZFNs [23], [40], [41].

**Figure 3:**
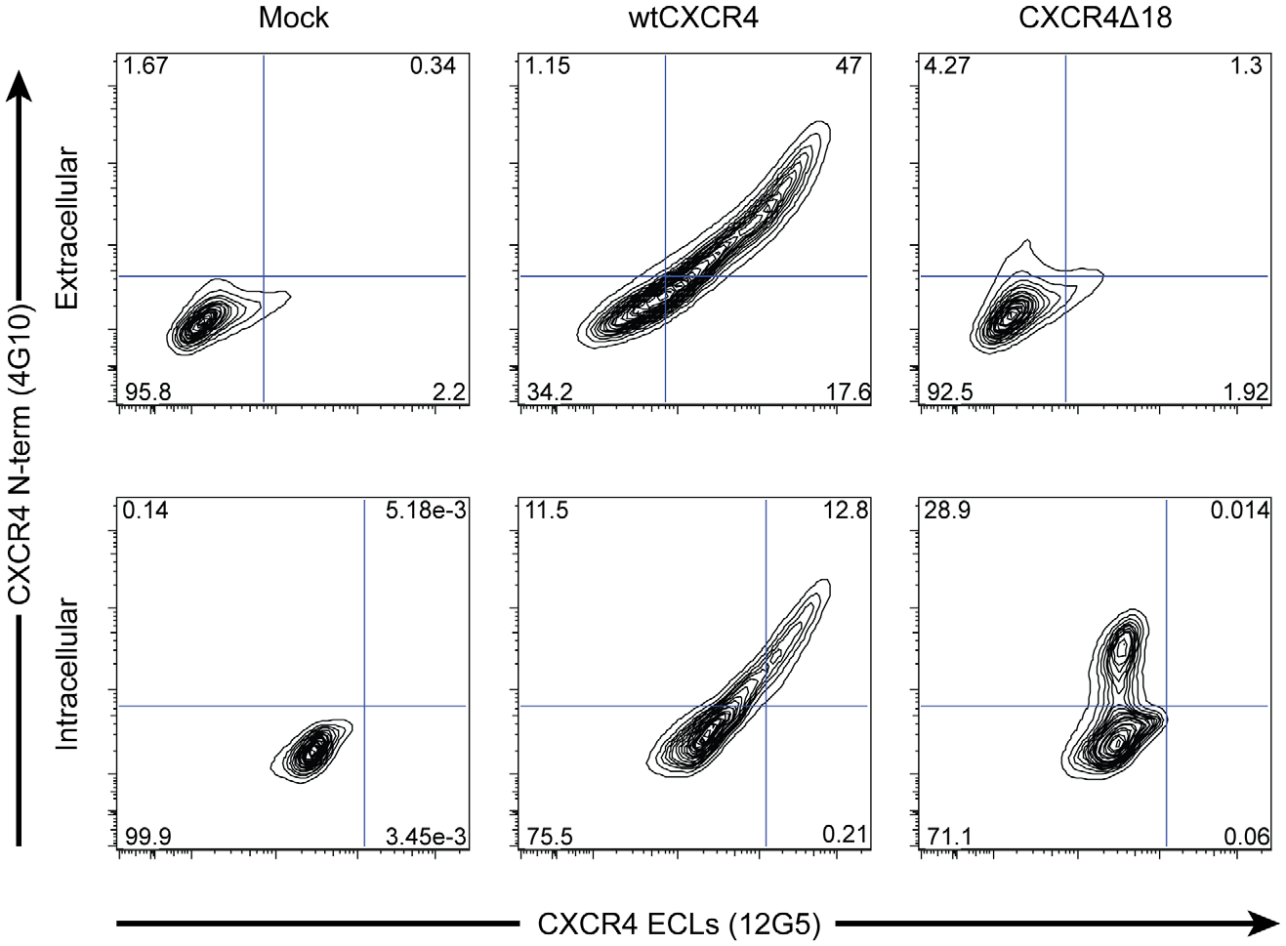
X4-ZFNs preferentially generate in-frame deletions resulting in the absence of CXCR4 cell surface expression. The most common lesion induced by the X4-ZFNs was an 18 bp deletion, *cxcr4Δ18*, that results in deletion of the amino acid sequence RFYPND from the second extracellular loop of CXCR4 (see Figure 1B). To determine if CXCR4Δ18 was expressed on the cell surface, a mock, wild type *cxcr4*, or *cxcr4Δ18* plasmid was transiently transfected into 293T cells that have low endogenous CXCR4 expression. Cells were then analyzed by flow cytometry after being stained simultaneously with anti-CXCR4 clone 4G10, which recognizes the N-terminus, and clone 12G5 whose epitope includes the second extracellular loop that is disrupted by the X4-ZFNs. WtCXCR4 was detected equally by both antibodies on the cell surface (middle panel, top row) and intracellularly (middle panel, lower row). However, CXCR4Δ18 was not detected by the N-terminal antibody on the cell surface (right panel, top row), but was detected when cells were permeabilized (right panel, bottom row) suggesting the 18 bp deletion prevents its expression on the cell surface.

To further characterize the consequences of disruption mediated by X4-ZFNs, we analyzed an unusually common lesion, an in-frame 18 bp deletion (CXCR4Δ18) that results in the deletion of DNA encoding amino acids R188 to D193 (Figure 1B). This deletion comprised 11.2% (range 9.8 and 11.9%) of all *cxcr4* disruptions across five independent experiments with cells from five different donors. The resulting CXCR4Δ18 protein, containing a six-residue deletion in ECL2, could potentially be expressed at the cell surface and support HIV infection. To examine this, we transiently expressed CXCR4Δ18 or wt CXCR4 as a control in 293T cells, which have low endogenous CXCR4 expression. CXCR4 cell surface and intracellular expression was detected by flow cytometry after co-staining with the N-terminal specific CXCR4 antibody 4G10 and the extracellular loop (ECL) specific antibody 12G5 whose epitope includes the CXCR4Δ18 deleted residues [42]. As expected, CXCR4 could be detected on the surface of control cells by both the N-terminal and ECL antibodies. However, CXCR4Δ18 was not detected at the cell surface, though it was detected intracellularly by the N-terminal antibody (Figure 3). In addition, cells expressing CXCR4Δ18 along with CD4 did not support HIV-1 infection. These findings indicate that CXCR4Δ18, the most common in-frame deletion resulting from the X4-ZFNs, does not readily traffic to the cell surface and does not function as an HIV-1 coreceptor.

### Specificity of cleavage by X4-ZFNs

Potential off-target genome modification comprises the predominant safety concern with ZFNs. Although ultra-deep full genome sequencing could best identify off-target effects, it is impractical and cost-prohibitive with current technology. Instead, we took a more targeted approach that used an experimentally derived binding site for each X4-ZFP to guide the identification of potential off-target cleavage sites. We conducted *in vitro* selection, or SELEX (**s**ystemic **e**volution of **l**igands by **ex**ponential enrichment) to determine the actual binding site preference of each X4-ZFP (Figure S1) [43], [44]. A positional-weighted matrix was then generated of the 12 bp binding site and 1 bp flanking region for each ZFP. A BLAST search against the human genome was then used to determine the top 15 off-target binding sites by allowing up to six mismatches per ZFP binding site, a 5 or 6 bp gap between ZFPs, and formation of hetero or homodimers (Table S2) [23]. To assess low frequency disruption events, we conducted 454 deep sequencing on all 15 sites in both control CD4+ T cells and those treated with X4-ZFNs, yielding approximately 7,500–26,000 reads per site in the ZFN-treated samples (Table S2). In a sample with 26.9% of CXCR4 alleles disrupted, NHEJ events were detected at a frequency of 2.3% (170/7531 reads) in an extragenic region on chromosome 12 and 0.8% (84/10531) in ADAMTS17, a metalloprotease of unknown function [45]. The four mutations out of 20,312 reads found in DEC1 (a putative tumor suppressor [46]) and the single mutation out of 21,139 reads found in an extragenic region of chromosome 11 could be due to PCR and sequencing errors or to very low levels (<0.02%) of ZFN-mediated cleavage events. Overall, the X4-ZFNs are highly specific for *cxcr4* with low frequency disruption clearly seen at 2 of 15 putative off-target sites with the highest homology to the intended target.

### X4-ZFNs confer *in vitro* protection to human CD4+ T cells from HIV challenge

Disruption of both *cxcr4* alleles should render human CD4+ T cells resistant to X4- and perhaps some R5X4- viruses as well, while cells harboring a single disrupted allele might express lower levels of CXCR4 and so be more resistant to virus entry. To determine whether ZFN-mediated disruption of *cxcr4* indeed protects CD4+ T cells from an *in vitro* HIV challenge, human CD4+ T cells from three different *ccr5* wild type donors were stimulated and transduced with AdX4-ZFNs or an AdR5-ZFNs control. Four days post-transduction, the cells were infected with three diverse HIV-1 strains: BK132 (primary X4 HIV), HxB2 (lab-adapted X4 HIV), or R3A (primary R5X4 HIV). Approximately two weeks post-transduction the cells were restimulated with anti-CD3/anti-CD28 beads, and cultures were maintained for an additional two weeks.

**Figure 4:**
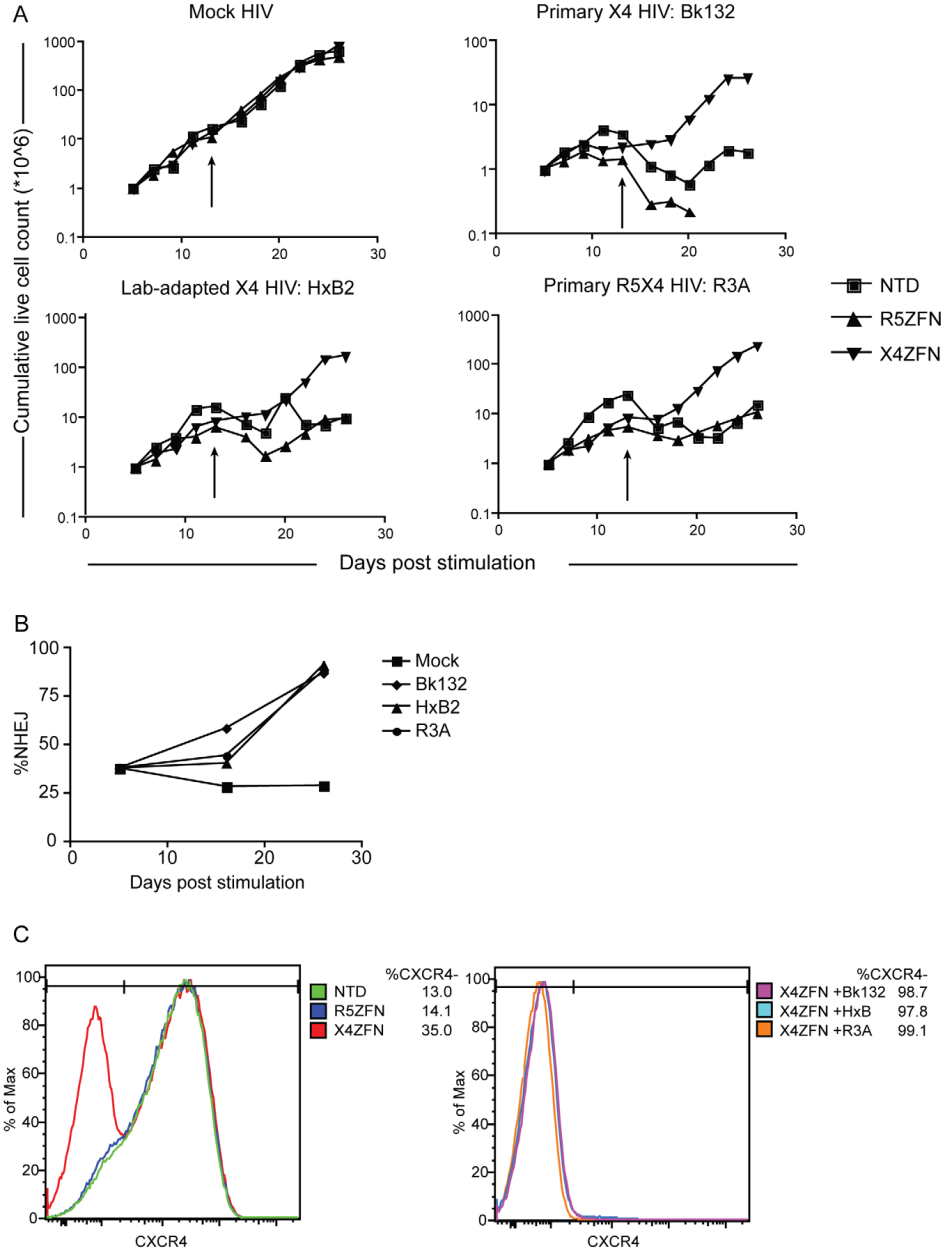
Treatment of human CD4+ T cells with X4-ZFNs confers protection to HIV-1 challenge *in vitro*. (A) Human CD4+ T cells were treated with the X4-ZFNs or R5-ZFNs expressed by Ad5/F35 vectors or were non-transduced (NTD). Four days later cells were infected with a primary X4 HIV-1 (Bk132), lab-adapted X4 HIV-1 (HxB2), primary R5X4 HIV-1 (R3A) or mock infected. The number of viable cells were measured at various times after stimulation. Cells were re-stimulated on day 13 (arrows). (B) The proportion of disrupted *cxcr4* alleles was determined at the indicated times post-stimulation by 454 deep sequencing. The frequency of *cxcr4* disruption was relatively constant in the mock-treated cells, but increased dramatically in the presence of HIV-1. (C) FACS analysis using a CXCR4-specific monoclonal antibody was performed at 19 days post infection (24 days post-stimulation). Mock HIV-infected cultures are shown on the left and HIV infected cultures on the right. Data shown is one of three independent experiments.

In the absence of HIV infection, there was no detectable growth difference between the X4-ZFNs treated, R5-ZFNs treated, and non-transduced controls over the course of the experiment. However, upon infection with the X4- or R5X4- HIV-1 strains, X4-ZFNs treated cells maintained exponential growth compared to profound cell death seen in the R5-ZFNs and untransduced controls. Despite the ability of R3A to utilize both CCR5 and CXCR4 to infect cell lines, in human CD4+ T cells stimulated with anti-CD3/anti-CD28 coated magnetic beads, CCR5 is downregulated causing transient resistance to R5 HIV [47]. Thus, R5X4 HIV strains are likely to function predominantly as X4 HIV strains under these conditions [47]. The growth advantage conferred by treatment with X4-ZFNs in the presence of HIV was magnified upon restimulation. (Figure 4A). This likely resulted from increased cell activation, which increases the ability of HIV to infect and replicate in CXCR4 positive cells.

To determine whether the growth advantage conferred by X4-ZFNs treatment in the presence of X4- and R5X4- HIV resulted from a survival advantage of CXCR4 disrupted cells, we performed flow cytometry at various time points post infection as well as deep sequencing of the X4-ZFNs target site on HIV-infected and uninfected cultures. In the absence of HIV infection, the *cxcr4* disruption frequency remained stable over time in four independent experiments testing four different *ccr5* wild type donors as measured by deep sequencing. A representative experiment is shown in Figure 4B and CXCR4 disruption data from all experiments is shown in Tables S1 and S3. While CXCR4 gene disruption remained stable over time at approximately 30%, CXCR4 gene disruption in HIV-infected cultures increased to 87%, 91%, and 88% in the presence of BK132, HxB2, and R3A respectively after 21 days of infection. FACS analysis showed that at day 19 post-HIV challenge, the frequency of CXCR4 negative cells amongst all live mock HIV-infected CD4+ lymphocytes was 13.0% in untransduced cells, 14.1% in cells transduced with R5-ZFNs, and 35.0% in cells transduced with X4-ZFNs compared to greater than 98%, 97%, and 99% of Bk132, HxB2, and R3A infected cultures transduced with the X4-ZFNs, (Figure 4C). We also found that after 19 days post-HIV infection, reduced but significant cell growth was detectable in several of the HIV-infected control cultures, untransduced and treated with R5-ZFNs. However, greater than 95% of these cells, compared to approximately 10% of cells treated with X4-ZFNs, were CD3+CD4- suggesting that the surviving cell population was protected from HIV infection by down-regulating CD4 (Figure S2). Thus, CXCR4 disruption had no impact on cell viability, but conferred a significant survival advantage in the presence of HIV strains that can use CXCR4 to infect cells. Furthermore, in control cultures that were untransduced or treated with R5-ZFNs, viral titers exponentially increased until extensive cell death began approximately 8–10 days post infection. In contrast, in cultures treated with X4-ZFNs viral titers steadily decreased after peak viremia while cell growth remained exponential suggesting there was not significant viral production (data not shown).

### *Ccr5*Δ*32* CD4+ T cells treated with X4-ZFNs are resistant to R5 and X4 HIV

**Figure 5:**
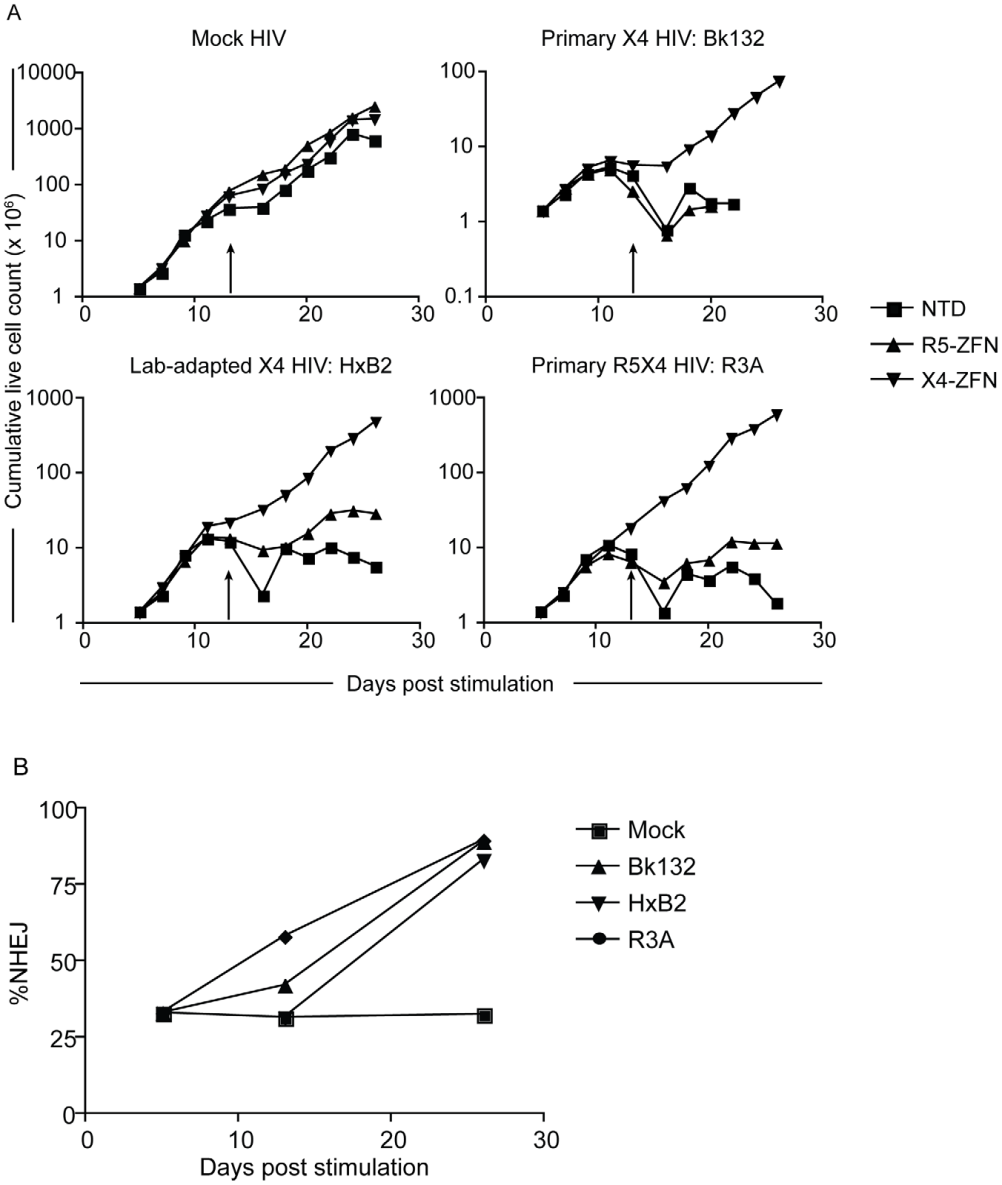
Treatment with X4-ZFNs is effective in *ccr5Δ32* homozgyous human CD4+ T cells. (A) *Ccr5Δ32* CD4+ T cells were stimulated on day 0 and transduced on day 1 with an Ad5/F35 vector expressing the X4-ZFNs, R5-ZFNs, or an untransduced control. On day 5, cells were HIV-infected with a mock, primary X4 HIV-1 (Bk132), lab-adapted X4 HIV-1 (HxB2), or a primary R5X4 HIV-1 (R3A). Live cells were counted approximately every two days. Cells were restimulated on day 13 (arrows). (B) *Cxcr4* disruption frequency was assessed at various times by 454 deep sequencing. Disruption remained stable in the absence of HIV-1 infection, but profoundly increased in the presence of the three HIV-1 strains examined. Data shown is from one of two representative experiments.

Given the ongoing adoptive therapy trial of CD4+ T cells treated with R5-ZFNs and the anti-viral success of the recent *ccr5Δ32* bone marrow transplant in an HIV-infected patient [6], we sought to determine if *cxcr4* could be genetically disrupted simultaneously with *ccr5*. Human CD4+ T cells from a *ccr5*Δ*32* homozygote were transduced with AdX4-ZFNs or AdR5-ZFNs and subsequently infected with HIV-1 strains Bk132, HxB2, and R3A as described above. Representative data from one of two independent experiments conducted in cells from the same donor is shown in Figure 5 and data from both experiments is shown in Tables S1 and S3. As seen in *ccr5* wild type CD4+ T cells, exponential cell growth was preserved in cultures treated with X4-ZFNs compared to control cultures that were untransduced or treated with R5-ZFNs (Figure 5A). In addition, disruption frequency in cultures treated with X4-ZFNs as determined by deep sequencing remained remarkably stable between 32–33% from day 5 to day 26 post-transduction in the absence of HIV, which suggests that simultaneous disruption of *ccr5* and *cxcr4* does not adversely affect cell growth. However, in the presence of Bk132, HxB2, and R3A, *cxcr4* disruption increased after 21 days of HIV challenge to 89%, 83%, and 90%, respectively (Figure 5B), and was associated with markedly diminished virus replication (data not shown), again consistent with significant protection conferred by *cxcr4* disruption. Thus, treatment with X4-ZFNs of both wild-type and *ccr5*Δ32 CD4+ T cells confers stable *cxcr4* disruption and a marked survival advantage in the presence of R5X4-HIV and X4-HIV *in vitro* without any detectable effect on cell growth or viability in the absence of HIV. This suggests that both *ccr5* and *cxcr4* can be genetically targeted simultaneously for the treatment of HIV infection, while preserving the replicative capacity of the CD4+ T cells.

### X4-ZFNs confer partial protection in NSG humanized mouse model

As a first step in evaluating the safety and efficacy of the X4-ZFNs *in vivo*, we employed a NSG humanized mouse model. Briefly, human CD4+ T cells were stimulated with anti-CD3/anti-CD28 beads and transduced with either AdX4-ZFNs or an AdR5-ZFNs control at an MOI of 600. Cells were then expanded *in vitro* for ten days after which 10^7^ CD4+ T cells treated with X4-ZFNs (n = 23) or R5-ZFNs (n = 22) were injected intravenously into each mouse. Engraftment was assessed by peripheral blood CD4+ T cell counts 27 days post-injection. All 45 animals successfully engrafted; however, one animal that received cells treated with the X4-ZFNs had a significantly higher but stable CD4+ T cell count and was thus excluded as an outlier from the remainder of the study. On day 28 post-engraftment, mice were intravenously injected with 10^5^ autologous CD4+ T cells that were previously infected with the highly cytopathic X4 HIV-1 strain Bk132 or a mock control. CD4 counts, viral load, and CXCR4 disruption were then monitored to determine the effect of treatment with X4-ZFNs.

**Figure 6:**
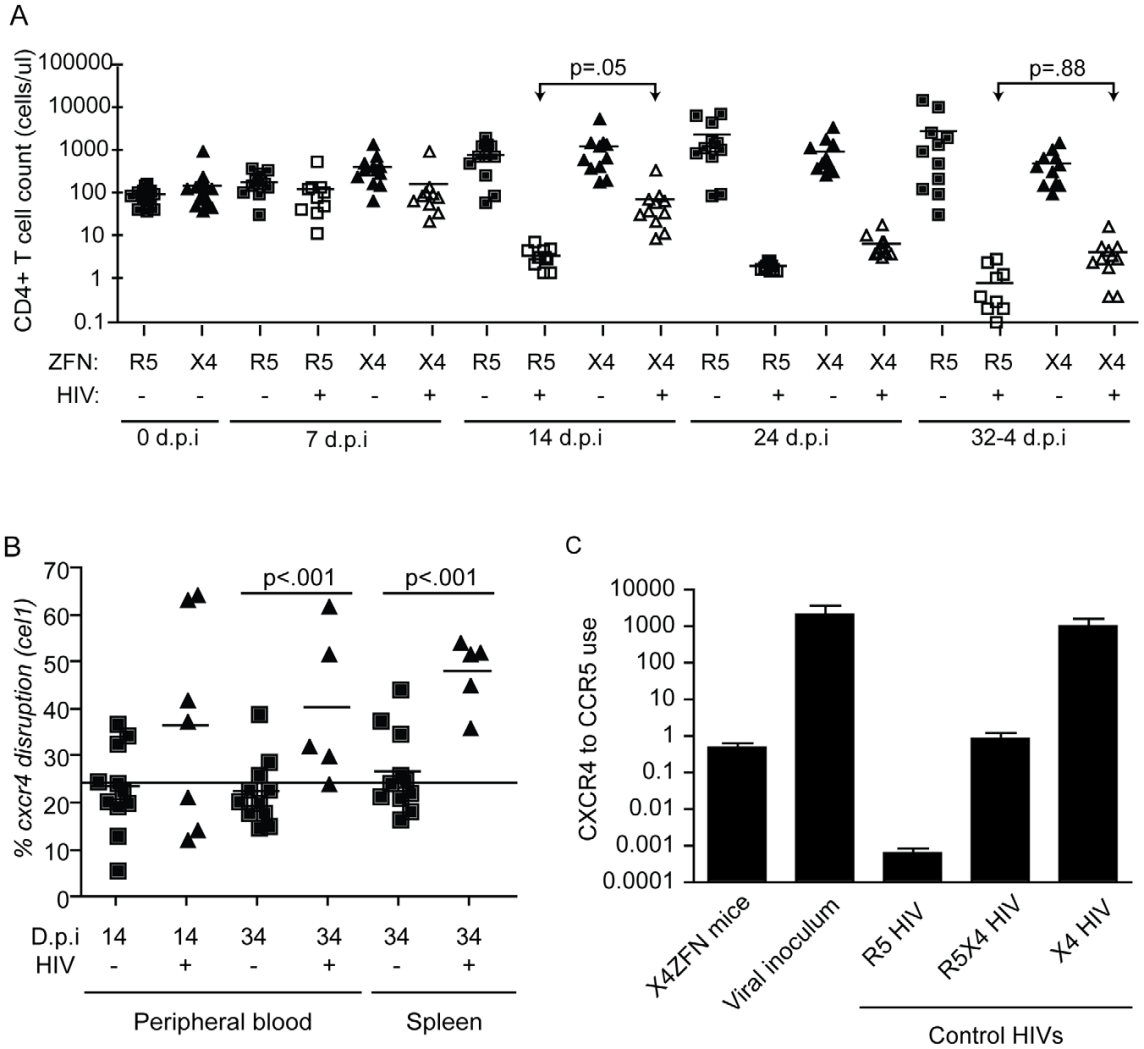
Treatment with X4-ZFNs confers partial protection to HIV-1 in humanized mice *in vivo*. NSG mice were injected with human CD4+ T cells treated with X4-ZFNs or R5-ZFNs. 28 days post injection, mice were infected with primary X4 HIV-1 (Bk132) or were mock-infected. (A) CD4+ T cell counts were measured every 7–10 days post infection. In the presence of Bk132, treatment with X4-ZFNs conferred protection at 14 d.p.i (p = .05); however, this protection wanes by 34 d.p.i. (p = .88) (B) *Cxcr4* disruption frequency was assessed by the surveyor nuclease assay in both peripheral blood (p<.001) and spleen (p<.001). At day 34 post infection, human CD4+ T cells were purified by positive selection prior to analysis to reduce any bias from low frequency contaminating human cells. Only samples with a detectable PCR signal are shown. Disruption frequency did not deviate significantly from the cell innoculum in either the blood or spleen. Data in (A) and (B) were analyzed by a general estimating equation (GEE). (C) HIV-1 Env from X4-ZFN mouse plasma was sequenced revealing a consensus Y302N mutation. To evaluate coreceptor tropism, a representative Env from the X4-ZFN mice and the viral innoculum were pseudotyped and used to infect NP2 cell lines expressing CD4 and either CCR5 or CXCR4. R5 HIV-1 (JRFL), R5X4 HIV-1 (R3A), and X4 HIV-1 (TYBE) controls are shown. Infectivity on NP2/CD4/CXCR4 cells was divided by that on NP2/CD4/CCR5 cells to determine relative coreceptor use. Data is an average of three independent experiments each done in triplicate. Error bars represent standard error.

To determine if X4-ZFNs impacted cell growth or viability in the absence of HIV, we first compared CD4 counts over time between the uninfected X4-ZFN and R5-ZFN control mice. There was no significant difference in CD4 counts between the two groups over the course of the 61 day experiment as determined by a generalized estimating equation (GEE) method (p = .88) (Figure 6A). Next, we examined the frequency of CXCR4 DNA disruption over time with the surveyor nuclease assay. At the time of injection the percentage of *cxcr4* alleles disrupted was 24.3%. This remained constant in both the blood (p = .32) and spleen (p = .70) over the course of the experiment suggesting that CXCR4 disruption did not significantly impact trafficking between these two compartments (Figure 6B). Next, we characterized CXCR4 cell surface expression over time by FACS. In the R5-ZFN control group, with intact *cxcr4* genes, 88% of CD4+ T cells expressed CXCR4 protein at day 27 post engraftment, compared to 84% of cells in the X4-ZFN mice (∼24% *cxcr4* gene disruption) as determined by a fluorescence minus-one (FMO) control. This difference persisted over time in the absence of HIV-1 infection (p <0.001) (data not shown). Together the stable disruption of CXCR4 as determined by both the surveyor nuclease assay and flow cytometry suggests that CXCR4 disruption did not negatively impact cell viability or growth in humanized NSG mice over a two-month period. As expected, xenogeneic graft versus host disease (GVHD), assessed clinically by dermatitis and hair loss, was observed in mice receiving cells treated with both R5-ZFNs and X4-ZFNs in the absence of HIV challenge. The development of GVHD was equivalent between the two groups (data not shown), suggesting that treatment with X4-ZFNs did not affect CD4+ T cell effector functionality.

In response to X4 HIV challenge with HIV-1 Bk132, CD4 counts decreased in both X4-ZFN and R5-ZFN mice. However, this rate of decline was slower in the X4-ZFN mice. The X4-ZFN group exhibited a mean 1.1 log CD4 count protection by day 14 post infection (p = .05 for a parametric t-test). However, this protective effect waned over time and there was no significant difference in CD4 counts by day 33 post infection (p = .88) suggesting that treatment with X4-ZFNs conferred only transient protection (Figure 6A).

One mechanism that could account for this would be if mutations arose in the viral Env protein to enable it to use CCR5. To explore this possibility, we bulk cloned and sequenced the V3 loop of Env, the main determinant of coreceptor tropism [48], from plasma isolated from three R5-ZFN mice and three X4-ZFN mice at the time of sacrifice. We identified a single amino acid substitution (Y302N) present in Env isolated from X4-ZFN mice but not R5-ZFN mice or the viral innoculum. Next, we cloned six distinct, functional Envs from the X4-ZFN mice and three distinct, functional Envs from the viral innoculum. As full length Bk132 Env would not pseudotype on an NL43 HIV core we truncated the cytoplasmic tail of the Envs [49], [50], and conducted tropism testing on NP2 cell lines expressing CD4 with either CCR5 or CXCR4. Of the six functional Envs from X4-ZFN mice, four contained the Y302N mutation. Interestingly, these four Envs were able to utilize CCR5 and CXCR4 equivalently, similar to the R5X4-tropic control R3A. All clones with the wild type Tyr302, including the Envs from the viral innoculum and two Envs from X4-ZFN mice utilized CXCR4 approximately 1000-fold more efficiently than CCR5 and comparably to the X4-tropic control TYBE (Figure 6C). Thus, in an NSG humanized mouse model of HIV infection, the cells treated with X4-ZFNs engrafted, trafficked, and persisted comparably to control cells. In addition, treatment with X4-ZFNs resulted in significant transient protection of CD4+ T cell counts in response to X4-tropic HIV challenge, and HIV challenge provided *cxcr4* disrupted cells with a survival advantage as determined by increase of *cxcr4* disruption in the presence but not the absence of HIV. However, the extent of the protection conferred by the X4-ZFNs was mitigated by evolution or outgrowth of preexisting R5X4-tropic HIV.

### ZFN-mediated coreceptor disruption is feasible in rhesus macaque CD4+ T cells

**Figure 7:**
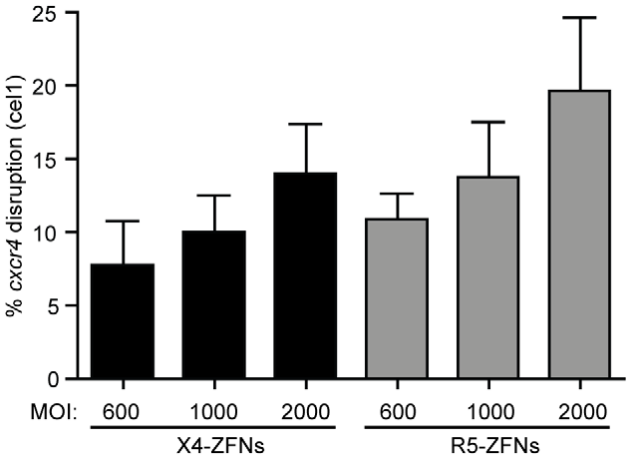
ZFNs can efficiently disrupt *ccr5* and *cxcr4* in rhesus macaque CD4+ T cells. The X4-ZFN pair's 24 bp binding site is conserved between humans and rhesus macaques. However, the human and rhesus R5-ZFNs have different binding sites; thus, a novel CCR5-ZFN pair was generated targeting rhesus *ccr5*. The rhesus R5-ZFNs and X4-ZFNs were delivered by Ad5/F35 vector at MOIs from 600–2000 into rhesus CD4+ T cells. Disruption frequency was measured by the surveyor nuclease assay. Data shown is an average of three independent experiments in cells from two different animals. Error bars represent standard error.

While humanized mouse models for HIV infection have utility, the model is limited due to incomplete immune reconstitution, development of xenogeneic graft versus host disease (GVHD), and the absence of normal T cell homeostasis. For these reasons and others, the NSG model is suboptimal compared to non-human primate models to further elucidate the safety and efficacy of treatment with X4-ZFNs and R5-ZFNs. As a proof of concept for future clinical adoptive therapy studies, we attempted to disrupt the *ccr5* and *cxcr4* genes with ZFNs in rhesus macaque CD4+ T cells. Briefly, rhesus CD4+ T cells were isolated from whole blood, purified by magnetic bead negative selection, and then stimulated with anti-CD3/anti-CD28 coated beads as previously described [35], [36]. As the 24 bp X4-ZFPs' binding site is identical between rhesus and humans, we were able to utilize the same ZFN pair. However, in order to target rhesus CCR5, rhesus specific R5-ZFNs were developed. As for human cells, the ZFNs were delivered with an Ad5/F35 vector and disruption was assessed by the surveyor nuclease assay. Utilizing a range of MOIs of 600, 1000, and 2000 we observed mean *ccr5* and *cxcr4* disruption levels of 19.6% and 14.0%, respectively (Figure 7), which suggests that adoptive therapy of cells modified with ZFNs is feasible to model in rhesus macaques.

## Discussion

The apparent eradication of HIV resulting from a *ccr5Δ32* homozygous allogeneic bone marrow transplant into an HIV-infected patient represents the first reported “cure” of HIV [6]. While an important proof-of-principle, few individuals could benefit from allogeneic *ccr5Δ32* homozygous transplants due to toxicities of allogeneic rejection and limitations of finding sufficient HLA-matched *ccr5Δ32* homozygous donors. However, coreceptor-specific ZFNs represent a novel therapeutic approach to recapitulate this success via autologous transplantation of gene-modified hematopoietic stem cells and mature CD4+ T cells. *Ccr5* can be efficiently disrupted in both human CD4+ T cells and hematopoietic stem cells, conferring protection to HIV challenge *in vitro* and in humanized mice [23], [28]. In addition, transgenic autologous hematopoietic stem cells can be successfully transplanted in HIV-infected individuals [18] and several phase I adoptive transfer trials of CD4+ T cells treated with R5-ZFNs in HIV infected individuals are currently underway. By design, this strategy addresses only viruses that require CCR5 to infect cells. Our long-term goal, therefore, is to explore the potential to genetically disrupt both *ccr5* and *cxcr4* for cell replacement therapies in HIV infected individuals, and in the case of *cxcr4* do so in a way that specifically targets CXCR4 on T cells and not the many other cell types on which it is expressed.

Unlike for *ccr5*, there are no known humans with loss of function *cxcr4* mutations that would provide insight into the safety and viability of *cxcr4* disruption in mature CD4+ T cells. A concern associated with targeting CXCR4 is that it is broadly expressed, while CCR5 expression is largely limited to hematopoietic cells. CXCR4, along with its natural ligand CXCL12, plays a critical role in normal B cell, cardiovascular, and cerebellar development, though T lymphocytes appear to develop normally in *cxcr4−/−* mice [51]. Thus, it is possible that the selective disruption of *cxcr4* in mature post-thymic CD4+ T cells may be tolerable. In addition to its role in development, the CXCR4-CXCL12 axis is a potent CD4+ T cell chemoattractant, and the broad expression of both proteins suggests that this axis may play a fundamental role in basal chemotaxis as opposed to a response to inflammation [52]. Indeed, inhibiting CXCR4 function systemically with the small molecule antagonist plerixafor results in the peripheral mobilization of hematopoetic stem cells, thus mitigating the potential of such therapy for long-term anti-retroviral therapy. However, plerixafor, which has not been reported to have adverse immunologic consequences resulting from inhibiting CXCR4 function in mature CD4+ T cells, provides proof of principle that inhibiting CXCR4 in mature CD4+ T cells may prove to be safe and viable [10], [53]. This suggests that this essential gene can be targeted in a cell-type specific manner with CXCR4-specific ZFNs that limits the toxicities of systemic disruption. While we have demonstrated that CXCR4 is not essential for CD4+ T cell viability and function *in vitro* and in humanized mice *in vivo*, the redundancy of lymphocyte chemokine receptors and their ligands makes predicting the *in vivo* consequences of *cxcr4* disruption in a normal host on CD4+ T cell function and trafficking difficult. We conclude that a logical next step will be to study the consequences of *cxcr4* disruption in a non-human primate model of HIV infection, which will simultaneously permit the assessment of the consequences of this approach on T cell function and trafficking.

A significant advantage of ZFN gene modification, compared to retrovirus based approaches, is that only transient transgene expression is required to permanently engineer an HIV resistant cell. As a result, adenovirus or other delivery mechanisms such as RNA transfection can be employed that avoid toxicities that can be associated with retroviral integration, such as cellular expansion or transformation. This “hit-and-run” approach limits the requirement of chronic transgene expression and the potential leakiness of other approaches including siRNA [21], [22], intrabodies [19], and ribozymes [17]. However, like most gene transfer approaches a major concern with ZFN technology is the potential for oncogenesis due to off-target effects. While additional study is clearly needed, our current studies have clearly identified off-target disruption in two of the top 15 putative off-target sites: an extragenic site on chromosome 12 and in the metalloprotease ADAMTS17, which is not expressed in CD4+ T cells. In addition, mature CD4+ T cells appear to be resistant to malignant transformation [54], thus mitigating the potential concerns of off-target disruption. Consistent with this, more than 200 people have safely undergone adoptive transfer of genetically engineered lymphocytes with no reported cases of therapy-induced oncogenesis [55]. Reasons for resistance to transformation of mature lymphocytes are unclear, but may involve an unknown mechanism that ensures the diversity of the TCR repertoire and thus limits clonal outgrowth [54]. In contrast, the safety record of hematopoietic stem cell gene therapy is less clear, with a significant frequency of gene-therapy induced oncogenesis or clonal outgrowth reported in several hematopoietic stem cell trials [56], [57].

One unexpected finding reported here is the predominance of in-frame mutations, particularly in-frame deletions, resulting from ZFN mediated cleavage of *cxcr4*. This has not been observed in other ZFN studies reported thus far. The deep-sequencing approach we have taken makes it possible to comprehensively and accurately assess the types and frequencies of mutations that result from ZFN cleavage followed by DNA repair. The striking preponderance of in-frame deletions may have resulted from toxicities of frameshift mutations shortly after treatment with X4-ZFNs leading to decreased survival relative to in-frame mutants. However, this is unlikely given that the frequency of in-frame mutations remained stable over nearly four weeks in culture, that there was no significant increase in cell death between control cultures and those treated with X4-ZFNs, and that the most common in-frame mutant was not expressed on the cell surface and thus cannot maintain functionality. Rather, the preference for in-frame deletions is likely due to preferential in-frame DNA repair. The deletion in the most common X4-ZFN-induced lesion, *cxcr4Δ18*, is flanked by a GTCA microhomology domain at the 5′ and 3′ ends consistent with a repair mechanism of microhomology-mediated NHEJ [58]. Similar microhomology sites are present in other common ZFN-induced *cxcr4* mutants that we identified. Thus, it appears that the nucleotide sequence of the X4-ZFN binding site directs a preference for an in-frame repair mechanism.

Our studies provide a fundamental demonstration that inactivation of *cxcr4* by treatment with X4-ZFNs rendered human CD4+ T cells resistant to infection by X4 virus strains, while CXCR4 inactivation in the context of a *ccr5Δ32* homozygous background rendered cells resistant to infection by both R5 and R5X4 strains. Genetic ablation of both CCR5 and CXCR4 will likely make CD4+ T cells entirely resistant to HIV-1. Dual-disruption of CCR5 and CXCR4 will be needed for maximal therapeutic benefit since 46% of treatment-experienced individuals harbor R5X4 strains of HIV compared to 4% with only X4-HIV strains [59]. While virus strains have been identified that can infect cells in the absence of CD4 (reviewed in [60]), none have been identified that can infect cells in the absence of a suitable coreceptor. In addition, virus strains that can use coreceptors other than CCR5 or CXCR4 to infect primary human cells are exceedingly rare. However, targeting CXCR4 alone could provide a selective advantage to CCR5-tropic virus strains. Suppression of CXCR4 by plerixafor *in vitro* can lead to the emergence of CCR5-tropic virus strains [61], and highly active antiretroviral therapy can sometimes result in enhanced prevalence of R5 relative to R5/X4 virus strains in infected patients [62]. In the humanized mouse model under the conditions studied here, partial loss of *cxcr4* in human T cells due to treatment with X4-ZFNs provided selective pressure for either the evolution or emergence of a pre-existing single amino acid mutation in the V3 loop of the infecting X4 HIV-1 strain that enabled it to use CCR5 as efficiently as CXCR4. Thus, just as either genetic or therapeutic suppression of CCR5 can provide an advantage to virus strains that use CXCR4, deletion of CXCR4 is expected to provide an advantage to CCR5-tropic viruses. However, this could provide a clinical benefit given the increased *in vitro* pathogenicity and correlation with progression to AIDS of X4-tropic HIV.

While humanized mouse models provided a logical first approach to examine *in vivo* efficacy of CXCR4 disruption, this system does not make it possible to fully assess the functional impact of CXCR4 loss on CD4+ T cell function. To study this in the most rigorous way possible, we have explored the possibility of targeting CCR5 and CXCR4 in CD4+ T cells derived from rhesus macaques. Following re-design of the R5-ZFNs to account for sequence differences between the human and macaque alleles, we found that ZFNs could disrupt both alleles with reasonable efficiency in macaque CD4+ T cells. By inactivating CXCR4 singly and in combination with CCR5, it will be possible to study the effects of CXCR4 loss on T cell function as well as virus infection in a more relevant animal model.

## Supporting Information

Figure S1 The DNA binding preference of the X4-ZFP left and X4-ZFP right was determined empirically by systemic evolution of ligands by exponential enrichment (SELEX). Briefly, a random pool of oligonucleotides was mixed with each ZFP. Unbound oligos were washed and bound oligos were amplified. After four rounds of selection, the enriched oligo pool was sequenced, and a position weighted matrix was generated for the 12 bp target site and one flanking residue per side (faded). Nucleotides corresponding to the wild type *cxcr4* sequence are shown above the horizontal line.
(TIF)

Figure S2 Treatment with X4-ZFNs prevents CD4 downregulation by HIV-1. CD4 is profoundly downregulated on live CD3+ cells HIV-1 infected cultures that were NTD or treated with R5-ZFNs but not X4-ZFNs. Thus, the limited cell growth remaining by 19 days post infection in NTD cultures and those treated with R5-ZFNs is due to HIV-1 induced CD4 downregulation, and thus the protective effect on cell growth for CD3+CD4+ cells is underestimated by the growth curves in Figure 4A. Cells are from same experiment as Figure 4.
(TIF)

Table S1 Deep sequencing results of *cxcr4* disruptions.
(XLS)

Table S2 Putative off-target sites of X4-ZFNs.
(XLS)

Table S3 Surveyor nuclease data after treatment with X4-ZFNs and challenge by HIV-1.
(XLS)
