# Supplementary figures and images for: Engineering HIV-Resistant Human CD4+ T Cells with CXCR4-Specific Zinc-Finger Nucleases
Source: PLoS Pathog. 2011 Apr 14;7(4):e1002020. doi: 10.1371/journal.ppat.1002020 (PMC3077364; doi:10.1371/journal.ppat.1002020)

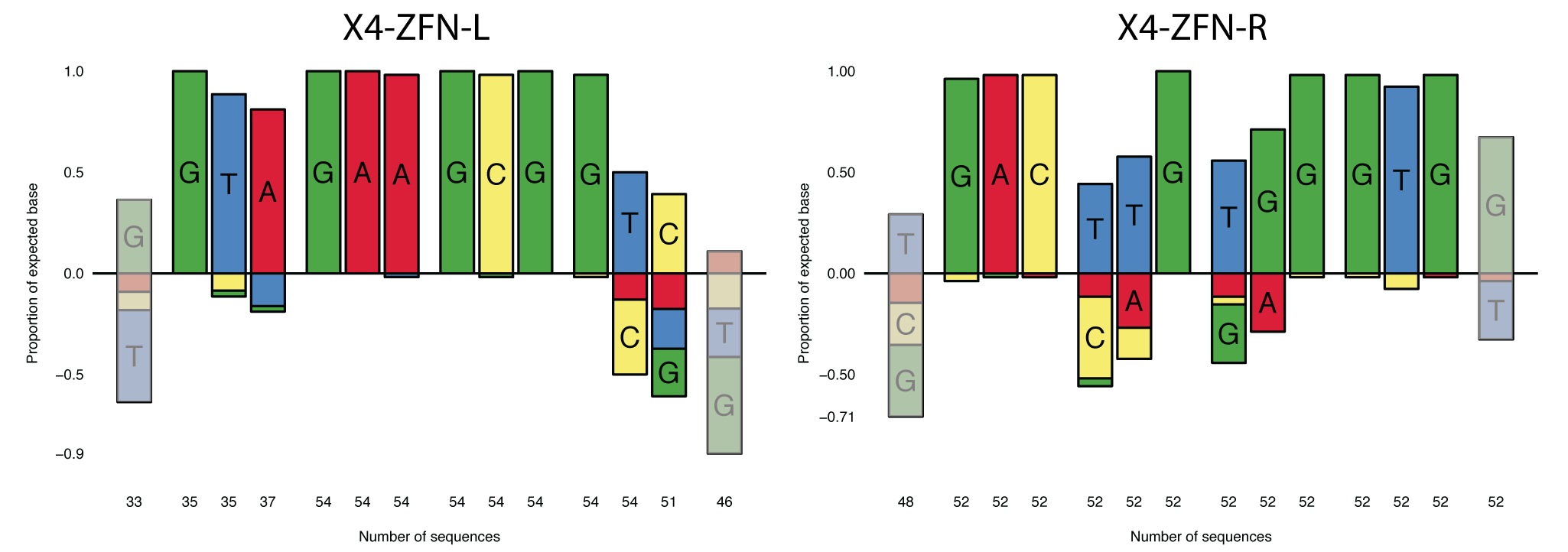

Supplement: Figure S1 — The DNA binding preference of the X4-ZFP left and X4-ZFP right was determined empirically by systemic evolution of ligands by exponential enrichment (SELEX). Briefly, a random pool of oligonucleotides was mixed with each ZFP. Unbound oligos were washed and bound oligos were amplified. After four rounds of selection, the enriched oligo pool was sequenced, and a position weighted matrix was generated for the 12 bp target site and one flanking residue per side (faded). Nucleotides corresponding to the wild type cxcr4 sequence are shown above the horizontal line. (TIF) [file ppat.1002020.s001.tif]

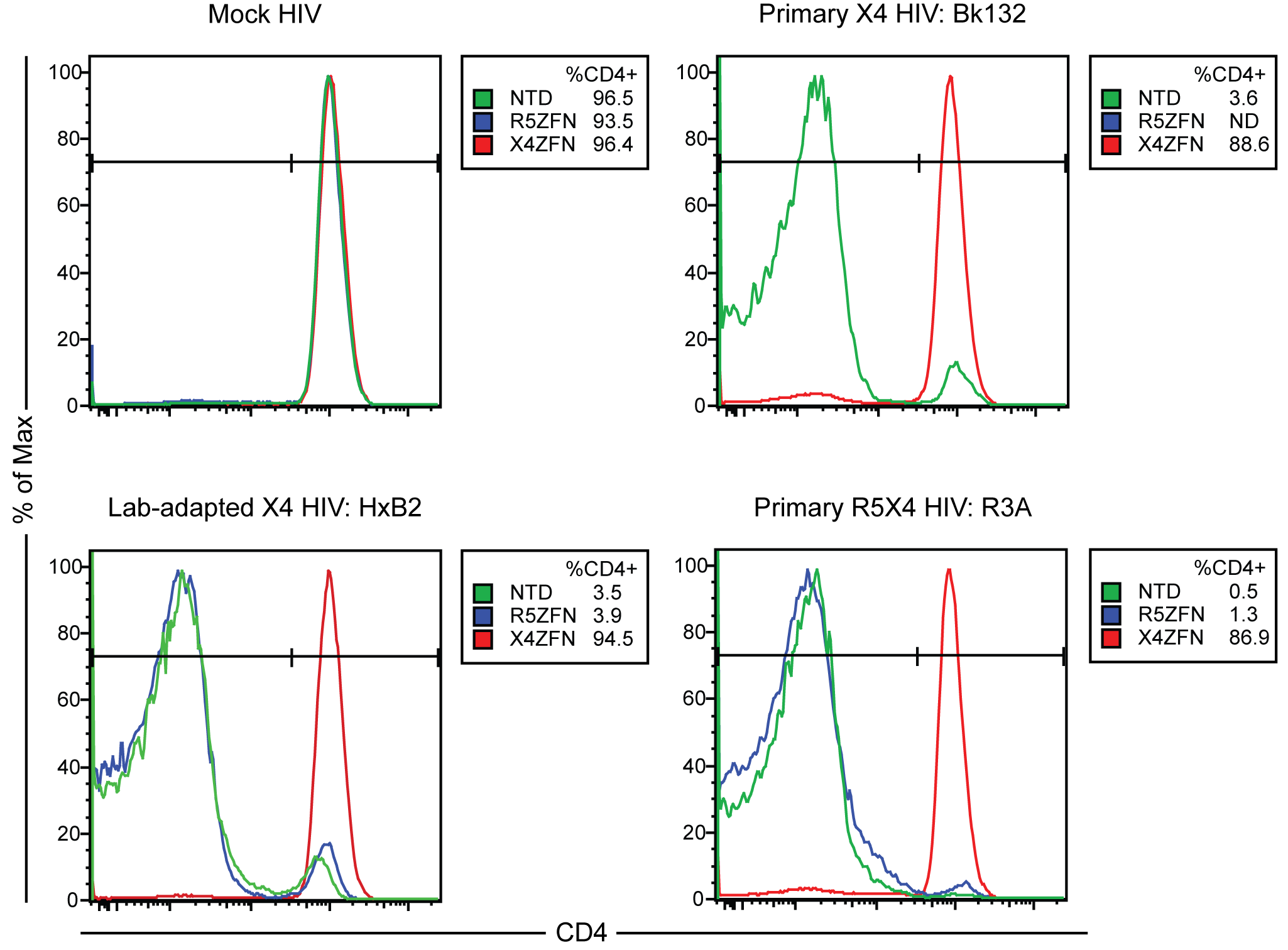

Supplement: Figure S2 — Treatment with X4-ZFNs prevents CD4 downregulation by HIV-1. CD4 is profoundly downregulated on live CD3+ cells HIV-1 infected cultures that were NTD or treated with R5-ZFNs but not X4-ZFNs. Thus, the limited cell growth remaining by 19 days post infection in NTD cultures and those treated with R5-ZFNs is due to HIV-1 induced CD4 downregulation, and thus the protective effect on cell growth for CD3+CD4+ cells is underestimated by the growth curves in Figure 4A. Cells are from same experiment as Figure 4. (TIF) [file ppat.1002020.s002.tif]
